# Selective Enzymatic Synthesis of Glucose Fatty Acid Esters in a Choline Chloride–Glucose Reactive Deep Eutectic Solvent

**DOI:** 10.1002/cssc.70963

**Published:** 2026-08-16

**Authors:** Jeong Eun Cha, Dojin Kim, Dong Han Kim, Keehoon Won, Sang Hyun Lee

**Affiliations:** ^1^ Advanced Materials Program Department of Biological Engineering Konkuk University Seoul Republic of Korea; ^2^ Department of Chemical and Biochemical Engineering Dongguk University‐Seoul Seoul Republic of Korea

**Keywords:** glucose ester, lipase, reactive deep eutectic solvent, transesterification

## Abstract

Sugar fatty acid esters are bio‐based surfactants, but their enzymatic synthesis is limited by poor substrate compatibility in conventional media. In this study, a choline chloride (ChCl)–glucose reactive deep eutectic solvent (RDES) was investigated for lipase‐catalyzed transesterification of glucose with vinyl laurate. In this system, glucose, choline, and water compete for a common acyl–enzyme intermediate, so product distribution is governed primarily by kinetic competition rather than thermodynamic equilibrium. By systematically controlling water content and the ChCl:glucose ratio, the effects of RDES composition on this pathway competition and product distribution were evaluated. Controlled adjustment of these parameters suppressed hydrolysis and competing choline‐derived ester formation, thereby promoting the selective formation of glucose monolaurate (GML). Under optimized conditions, a GML yield of approximately 46% was achieved, corresponding to a volumetric productivity of 12 g L^−1^ h^−1^, which significantly exceeded that obtained in conventional organic solvent‐based systems. The synthesized GML was purified and structurally confirmed using high‐performance liquid chromatography, mass spectrometry, and ^1^H and ^13^C nuclear magnetic resonance. The applicability of the optimized RDES system was further demonstrated using vinyl esters with varying acyl chain lengths, highlighting its broader potential for selective enzymatic synthesis of sugar fatty‐acid esters.

## Graphical abstract

Choline chloride–glucose reactive deep eutectic solvents direct lipase‐catalyzed acyl transfer toward glucose esters by restricting water‐ and choline‐mediated side reactions. Control of water content and solvent composition enables selective glucose monolaurate synthesis at high substrate loading, delivering a volumetric productivity of 12 g L^−1^ h^−1^.

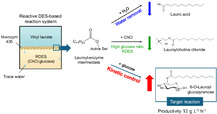

## 1 Introduction

Surfactants are widely used as surface‐active agents and emulsifiers in diverse industries, including food, cosmetics, pharmaceuticals, and detergents [1, 2]. In particular, food‐grade surfactants play essential roles in emulsion stabilization, texture modification, and shelf‐life extension in processed foods [3]. However, most commercially available surfactants are synthesized from petroleum‐based feedstocks, which present inherent limitations such as poor biodegradability, toxicity, and reliance on nonrenewable resources [4]. Further, emissions generated throughout their life cycle contribute substantially to environmental burdens [5, 6]. These constraints have motivated sustained efforts to replace conventional petrochemical surfactants with alternatives derived from renewable resources.

Sugar fatty acid esters (SFAEs) are bio‐based, nonionic surfactants considered as potential substitutes for conventional surfactants [7]. Derived from renewable sugars and fatty acids, SFAEs exhibit low toxicity, biodegradability, and neutral sensory properties, which support their use as food‐grade emulsifiers and stabilizers [2, 6, 8]. In addition to their interfacial activity, certain SFAEs have been reported to possess antimicrobial and insecticidal activities [9, 10]. The amphiphilic molecular structure of SFAEs, comprising a hydrophilic sugar moiety and a hydrophobic aliphatic chain, allows systematic modulation of physicochemical properties by varying the sugar unit, alkyl chain length, and degree of saturation [10, 12]. Such structural flexibility provides opportunities to tailor interfacial behavior in diverse systems. However, their industrial applicability ultimately depends on the feasibility of production processes, particularly in terms of achieving both high selectivity and sufficient volumetric productivity.

On an industrial scale, SFAEs are typically synthesized via chemical routes because of their cost‐effectiveness and high yields [13]. However, these methods often suffer from undesired side reactions and poor regioselectivity. Considering sustainability, chemical processes are energy intensive and impose environmental burdens owing to the use of volatile organic solvents and acid catalysts [8]. Moreover, such processes are not always compatible with food‐grade manufacturing requirements. As an alternative, enzymatic synthesis using lipases has been extensively investigated because it enables regioselective esterification under comparatively mild reaction conditions [14]. Use of immobilized enzymes can further improve the process economics by enhancing operational stability and enabling catalyst reuse [15]. Despite these advantages, the enzymatic synthesis of SFAEs remains challenging because of the intrinsically low mutual solubility of hydrophilic sugars and hydrophobic fatty acids within a single reaction medium. Moreover, maintaining enzyme stability in hydrophilic organic solvents capable of efficiently dissolving sugars is inherently difficult [16, 17]. These limitations arise not only from catalytic performance but also from the reaction medium, which governs substrate accessibility and enzyme compatibility. Therefore, solvent selection repeatedly dictates the feasibility of enzymatic SFAE production, motivating the exploration of alternative nonaqueous reaction media that can simultaneously accommodate both substrates while avoiding toxic, flammable, and volatile organic solvents [18]. In particular, the low solubility of sugars in conventional media limits their concentration in the reaction phase, thereby limiting volumetric productivity, which remains a key barrier to the industrial implementation of enzymatic sugar ester synthesis.

To overcome the solvent‐related limitations associated with conventional organic media, ionic liquids (ILs) have been explored as alternative reaction media owing to their negligible vapor pressure and high thermal and chemical stability [13, 19]. In this context, our group has extensively investigated lipase‐catalyzed glucose ester synthesis in IL‐based systems. Through systematic studies on substrate solubility enhancement, water content control, and process optimization, we established IL‐based platforms enabling selective and high‐productivity enzymatic acylation of glucose as a model sugar [20, 23]. Process optimization in ILs revealed the critical role of water content in controlling enzyme activity and selectivity, while mixed IL systems were shown to further improve substrate availability and reaction performance [20, 22]. Additionally, process intensification strategies, including ultrasound‐assisted catalysis, were explored to enhance reaction rates and productivity [23].

However, despite these improvements, IL‐based systems still face limitations in terms of cost, recovery, and process complexity, which restrict their practical applicability in large‐scale production [13, 24]. As an accessible alternative, deep eutectic solvents (DESs) have been introduced and shown to exhibit physicochemical properties comparable with those of ILs [25, 26]. DESs are hydrogen‐bonded mixtures composed of a hydrogen bond acceptor (HBA) and a hydrogen bond donor (HBD), which remain liquid at temperatures far below the melting points of their individual components due to strong intermolecular hydrogen‐bonding interactions [27]. Commonly studied DES systems include choline chloride (ChCl) combined with urea, glycerol, or organic acids, many of which are composed of inexpensive and biocompatible components [28]. Their nonvolatility, relatively low toxicity, facile preparation, and ability to maintain enzyme stability under low‐water conditions have motivated their application in enzymatic transformations [24, 29, 31]. Such characteristics make DESs attractive media for developing enzymatic nonaqueous processes. Nevertheless, the sustainability of a DES‐based process cannot be inferred from the solvent class alone and should be evaluated in the context of energy input, reaction productivity, downstream solvent use, and opportunities for solvent and catalyst reuse.

More recently, reactive DESs (RDESs) have been proposed, in which one or more DES components simultaneously function as reaction media and reactive species, leading to their description as “2‐in‐1” systems [32, 34]. This concept provides an attractive approach to improving substrate availability in sugar‐based transformations. However, because solvent components can directly participate in acyl‐transfer reactions, product formation in RDES systems is governed by competing reaction pathways rather than by solubility alone. Several studies have investigated the enzymatic esterification of glucose using RDESs. Pöhnlein et al. reported the transesterification of glucose with vinyl hexanoate in ChCl:urea and ChCl:glucose formulations [35]. Notably, glucose‐containing DES enabled ester formation without externally added glucose, confirming its function as an RDES, while choline‐derived esters were observed as competing side products. In a subsequent study, Hollenbach et al. synthesized glucose decanoate in both hydrophilic and hydrophobic RDES systems and demonstrated that vinyl esters were generally more effective acyl donors than free fatty acids due to the equilibrium‐shifting effect of acetaldehyde formation [36, 37]. In contrast, Buzatu et al. reported that enzymatic esterification of glucose with lauric acid (LA) in ChCl:glucose:water RDES did not yield glucose esters, with lauroylcholine chloride (LCC) identified as the sole esterification product [38]. Their combined experimental and computational analyses attributed this unexpected selectivity to strong hydrogen‐bonding interactions between glucose and chloride ions, which were proposed to hinder the access of glucose to the catalytic site. These findings indicate that, without proper control of reaction conditions, ChCl–glucose RDES systems can favor competing pathways that lead to choline‐derived esters rather than the desired sugar esters.

Overall, RDES‐based sugar esterification is highly sensitive to acyl donor identity, water content, and DES composition, and varying these parameters can lead to changes in reaction efficiency and fundamentally different esterification outcomes, including the preferential formation of competing ester species [37, 39]. Taken together, these studies show that RDES composition influences not only substrate availability but also the relative rates of competing acyl‐transfer reactions. However, it has not yet been established whether changes in RDES composition can shift this competition in favor of sugar ester formation while limiting hydrolysis and choline‐derived ester formation. A systematic understanding of these effects is therefore needed for the rational design of RDES media for SFAE synthesis.

Accordingly, this study examined whether water content and the ChCl:glucose ratio could be used to control the competition among glucose, choline, and water for a common acyl–enzyme intermediate. Glucose laurate was selected as a representative sugar fatty acid ester for systematic investigation in a ChCl–glucose RDES system. The effects of water content and the relative ratio of ChCl to glucose were systematically examined by monitoring the formation of glucose ester, LA, and LCC as products of the competing pathways. The key reaction parameters were further optimized, and the resulting system was compared with conventional organic solvent‐based media and previously reported DES‐based approaches. The optimized strategy was subsequently extended to additional acyl donors to evaluate its broader applicability in SFAE synthesis. The results show that RDES composition and water content can be used to redirect competing acyl‐transfer pathways while maintaining high substrate availability for SFAE synthesis.

## 2 Materials and Methods

### 2.1 Materials

ChCl (99%), anhydrous glucose (98%), ethyl acetate (EtAc, 99.5%), sulfuric acid (95%), n‐hexane (95%), phosphoric acid (85%), methanol (99.9%), and high‐performance liquid chromatography (HPLC)‐grade water were purchased from Samchun Pure Chem. Co., Ltd. (Gyeonggi‐do, South Korea). 2‐Methyl‐2‐butanol (2M2B, 99%), vinyl laurate (VL, 99%), molecular sieve (3 Å), dimethyl sulfoxide‐d_6_ (DMSO‐d_6_, 99.9%), p‐nitrophenyl butyrate (pNPB) and glucose oxidase/peroxidase reagent were acquired from Sigma–Aldrich (St. Louis, MO, USA). Lauroylcholine chloride (LCC, 98%), vinyl hexanoate (99%), vinyl octanoate (99%), and vinyl decanoate (99%) were purchased from Tokyo Chemical Industry Co., Ltd. (Tokyo, Japan). DMSO (99%) was acquired from Kanto Chemical Co., Inc. (Tokyo, Japan), LA (99%) was obtained from Duksan Pure Chemicals Co., Ltd. (Gyeonggi‐do, South Korea), and sodium 1‐octanesulfonate (98%) was purchased from Daejung Chemicals & Metals (Gyeonggi‐do, South Korea). Lipase B from *Candida antarctica* (CALB) immobilized on acrylic resin (Novozym 435) was supplied by Daejongzymes Co., Ltd. (Seoul, South Korea). All chemicals were used as received without additional purification.

### 2.2 Preparation and Characterization of RDESs

ChCl–glucose RDESs consisting of ChCl and glucose were prepared in molar ratios of 2:1 (C2G1), 1:1 (C1G1), 1:2 (C1G2), 1:3 (C1G3), and 1:4 (C1G4). All RDESs contained the same amount of glucose (8 mmol). Each component was preweighed and dried overnight at 60 °C in a drying oven in the presence of silica gel. The dried compounds were then transferred to 20 mL screw‐capped vials, sealed, and stirred at 90 °C until a transparent liquid was obtained. The resulting RDESs were cooled to room temperature. The water content of the prepared RDESs was measured using a Karl Fischer titrator (870 KF Titrino Plus; Metrohm, Switzerland).

Thermal analysis was performed using a differential scanning calorimeter (DSC) (DSC8000, PerkinElmer, USA). Approximately 10 mg of each sample was placed in sealed aluminum pans and analyzed under dry nitrogen (99.99%), at a flow rate of 20 mL/min. The samples were subjected to first heating, cooling, and second heating cycles over the temperature range of −60 to 120 °C at heating rates of 20 °C/min. The samples were held isothermally for 10 min at the end of each heating and cooling step.

### 2.3 Hydrolytic Activity of Novozym 435 and General Procedure for Lipase‐Catalyzed Transesterification of Glucose in RDES

The hydrolytic activity of Novozym 435 was determined using the hydrolysis of pNPB according to a previously reported method [18]. Briefly, 10 mg of Novozym 435 was suspended in 9.5 mL of 0.1 M sodium phosphate buffer (pH 7.0), and the reaction was initiated by adding 0.5 mL of 10 mM pNPB dissolved in isopropyl alcohol. The mixture was incubated at 25 °C and 120 rpm, and 300 μL aliquots collected at 1‐min intervals for 10 min were mixed with an equal volume of acetonitrile before measuring the absorbance at 400 nm. One unit (U) of activity was defined as the amount of Novozym 435 that released 1 μmol of p‐nitrophenol per minute under the assay conditions. The measured activity of Novozym 435 was 11.8 ± 0.7 U/g biocatalyst; accordingly, the standard enzyme loading of 0.40 g corresponded to 4.7 U. This activity value was used to standardize the enzyme loading and does not represent the actual transesterification activity in the RDES medium. The enzyme was stored at 4 °C under controlled humidity in an airtight container with both silica gel and molecular sieves for at least 3 days prior to use.

**FIGURE 1:**
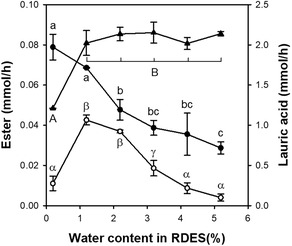
Reaction rates for glucose monolaurate (filled circles) and lauroylcholine chloride (unfilled circles) formation during the lipase‐catalyzed reaction of vinyl laurate in ChCl–glucose reactive deep eutectic solvent (RDES) (1:1 molar ratio of ChCl to glucose, C1G1) at various initial water contents. Formation rates of lauric acid (triangles) are shown on the right axis. Reaction conditions: C1G1 RDES (8 mmol ChCl and 8 mmol glucose), 8 mmol vinyl laurate, 0.4 g (4.7 U) enzyme, 125 rpm, 70 °C, 3 h. Reaction rates were calculated based on product formation within the fixed 3 h reaction period. Different letters indicate statistically significant differences (*p* < 0.05).

The approximate volumes of the C2G1, C1G1, C1G2, and C1G3 RDES phases containing 8 mmol of glucose were 3.13, 2.06, 1.47, and 1.32 mL, respectively. Except for the water‐content experiments shown in Figure 1, reactions were started by adding VL (8, 16, 24, or 32 mmol, corresponding to approximately 2.08, 4.16, 6.24, or 8.32 mL, respectively), along with 0.4 g of molecular sieves and Novozym 435 (0.27, 0.40, 0.54, 0.68, or 0.82 g), to a vial containing the RDES. The approximate initial liquid‐phase volume was 3.40–5.21 mL in the RDES‐composition experiments and 3.40–9.64 mL in the VL‐loading experiments. All components were introduced into the RDES in a moisture‐controlled glove box with silica gel. The vial was then sealed and placed in a silicone oil bath maintained at 70 °C on a stirring hot plate (Cimarec SP131010‐33, Thermo Scientific, USA) and stirred at 125 rpm. For the water‐content experiments shown in Figure 1, predetermined amounts of water were intentionally added to the C1G1 RDES before reaction initiation, and molecular sieves were not included so that the effect of the initially adjusted water content could be evaluated. In all subsequent experiments, no water was intentionally added, and molecular sieves were included to minimize residual and adventitious moisture.

Reactions were terminated at predefined time points according to the purpose of each experiment. Reaction rates were calculated from product formation measured after fixed reaction periods of 3 or 6 h, whereas product yields were evaluated after reaction periods of up to 24 h. For time‐course experiments, parallel reaction vials were independently terminated at the specified sampling times. The exact reaction times used in each experiment are provided in the corresponding figure captions.

### 2.4 Extraction and Purification of Glucose Ester

At the predefined reaction time, the vial was removed from the 70 °C oil bath and allowed to cool to approximately 40 °C. The immobilized enzyme and molecular sieves were not removed from the mixture before extraction to avoid any potential losses of substrates and products associated with these solid materials. A 5 mL portion of water was added to the reaction mixture, which was then homogenized in an ultrasonic bath (Powersonic 405, Hwashin Technology, South Korea) for 30 min. After all the components were mixed and a turbid liquid was formed, 10 mL of EtAc was introduced into the vial. Extraction was performed by vigorous stirring at 50 °C for 20 min. The upper hydrophobic phase was separated by centrifugation, filtered, and analyzed using HPLC for quantification of glucose ester and LA. The upper hydrophobic phase contained glucose ester, LA, LCC, and unreacted VL. In contrast, the lower hydrophilic phase contained unreacted glucose, unreacted ChCl, acetaldehyde, and a minor fraction of LCC. The concentrations of unreacted VL, glucose ester, and LA in the lower hydrophilic phase were below the detection limits. To evaluate whether additional reactions occurred during extraction, a 0 h control prepared under otherwise identical reaction conditions was immediately subjected to the same water addition, 30 min sonication, and EtAc extraction procedure. No glucose ester, LA, or LCC was detected in the 0 h control. In a separate control experiment using a reacted mixture containing a high concentration of glucose ester, the glucose ester content decreased by only approximately 1.1% during the same extraction procedure, indicating that additional reaction and glucose ester hydrolysis during extraction were negligible.

To purify the glucose ester, EtAc in the upper hydrophobic phase was evaporated under reduced pressure. The residue was washed three times with n‐hexane to remove unreacted VL and the produced LA. This washing step was used to obtain high‐purity glucose ester for structural characterization and preparation of an analytical standard. The precipitated water‐insoluble glucose ester was air‐dried, washed three times with deionized water to remove LCC, which was soluble in water, and then dried overnight at room temperature. The purity of the glucose ester obtained as a white powder was further characterized using HPLC, mass spectrometry (MS), and nuclear magnetic resonance (NMR); the compound was then used as the standard for 6‐O‐lauroyl glucose (glucose monolaurate, GML). Under the optimized C1G3 conditions, approximately 0.80 g of purified GML was obtained after 12 h, corresponding to an isolated yield of approximately 28% based on the initial amount of glucose.

### 2.5 Product Quantification Using HPLC Analysis

LA and GML produced in the upper hydrophobic phase were quantified by HPLC using a Nexera 40 Series system (Shimadzu, Japan) equipped with an Atlantis T3 C18 column (5 μm, 4.6 × 250 mm, Waters, Ireland) and an RI detector. The chromatographic conditions were adapted from a previously reported method with minor modifications [20]. The mobile phase comprised methanol and water (85:15, v/v) containing 0.01% (v/v) phosphoric acid. The flow rate was set as 1.0 mL/min with a total run time of 30 min, and the injection volume was 10 μL. Both the column and detector were operated at 40 °C. Under these conditions, GML and LA were eluted at 6.1 and 9.4 min, respectively (Figure S1). Quantitative analysis was performed using external calibration with purified GML and authentic LA standards. Calibration curves were constructed over a concentration range of 1–10 mg/mL, and the coefficients of determination (*r* ^2^) exceeded 0.999 for both analytes. Peak areas were used for quantification. Purified GML consistently exhibited two closely eluting peaks, which were attributed to the α‐ and β‐anomeric forms of 6‐O‐lauroyl glucopyranose. Therefore, the combined area of the two peaks was used for calibration and quantification. The corresponding calibration curves are provided in Figure S1. The product yield was calculated based on the theoretical maximum yield, defined by the initial amount of the limiting substrate (glucose), assuming complete conversion to the corresponding monoester, as shown in the following equation

(1) $$\text{Yield}\;(\%)=\frac{\text{amount of glucose monolaurate formed}\;(\text{mmol})}{\text{initial glucose amount}\;(\text{mmol})}\times 100$$

The reaction rate was calculated from the content of product formed per unit time as follows:

(2) $$\text{Reaction rate}\;\;\left(\text{mmol}/h\right)=\frac{\text{formed product amount}\;(\text{mmol})}{\text{time}\;(h)}$$

For LCC quantification, a separate parallel reaction vial was prepared and reacted under conditions identical to those used for GML and LA analysis. At the same predefined reaction time, 10 mL of 60% (v/v) methanol was added directly to this reaction mixture, followed by sonication for 30 min. The resulting cloudy suspension was centrifuged at 8,000 rpm for 10 min, and the transparent hydrophilic phase was collected and filtered for HPLC analysis. LCC was quantified according to a previously reported method with minor modifications [40]. The analysis was performed using a YL9100 HPLC system (Younglin, South Korea) equipped with a SunFire C18 column (5 μm, 4.6 × 150 mm, Waters, Ireland) and a UV detector. The mobile phase consisted of methanol and water (80:20, v/v) containing 15 mM phosphoric acid and 5 mM sodium 1‐octanesulfonate. The flow rate was set at 1.0 mL/min with a run time of 30 min. The column temperature was maintained at 40 °C, and detection was carried out at 210 nm. Under these conditions, LCC was eluted at 4.1 min (Figure S2). Quantitative analysis was conducted using external calibration with authentic LCC standards over a range of 0.3–10 mg/mL. The calibration curve showed a coefficient of determination (*r* ^2^) exceeding 0.999. The corresponding calibration curve is provided in Figure S2. Peak areas were used for quantification, and the reaction rate was calculated based on Equation (2).

### 2.6 Characterization of the Glucose Ester Using MS and NMR

Purified GML was characterized using MS and NMR spectroscopy. MS analysis was performed using a mass spectrometer (LTQ XL, Thermo Fisher Scientific, USA) in the positive ion mode with electrospray ionization (ESI) [35]. Samples were dissolved in methanol containing 0.1% formic acid and directly infused into a mass spectrometer. Nitrogen was used as the nebulizing gas, and the spectra were recorded over an *m*/*z* range of 100–2,000 (Figure S3). The expected molecular weight of the product was 362.5 Da.

For structural confirmation, purified GML was dissolved in 0.5 mL of DMSO‐d_6_ at a sufficiently high concentration to obtain adequate signal intensity for 13C NMR analysis [20]. ^1^H and ^13^C NMR spectra were recorded using a 400 MHz NMR spectrometer (Avance NEO 400, Bruker, Germany) (Figure S4). Chemical shifts (*δ*) are reported in ppm relative to the residual solvent signals of DMSO‐d_6_ (*δ*H 2.50 ppm; *δ*C 39.5 ppm). The ^1^H‐NMR spectrum (400 MHz, DMSO‐d_6_) exhibited the following signals (*δ* in ppm): 0.85 (t, 3H), 1.23 (m, 18H), 1.50 (m, 2H), 2.26 (t, 2H), 3.02–3.12 (m, 2H), 3.76 (m, 1H), 3.98–4.27 (m, 2H), 4.51 (m, 1H), 4.74–4.89 (m, 1H), and 5.02 (d, 1H). The ^13^C NMR spectrum (100 MHz, DMSO‐d_6_) exhibited signals at *δ* (ppm): 14.0, 22.2, 24.5, 28.3, 28.6, 29.0, 29.1, 31.4, 33.5, 63.9, 66.6, 69.2, 70.2, 70.6, 72.2, 72.9, 73.6, 74.7, 76.4, 92.3, 97.0, and 173.0.

### 2.7 Lipase‐Catalyzed Transesterification of Glucose With Various Vinyl Esters in RDES

To evaluate the applicability of various vinyl esters, glucose esterification reactions were carried out under the reaction conditions optimized for VL. The reaction procedure was identical to that previously described. Briefly, the reactions were conducted with C1G3 RDES (8 mmol glucose; ca. 1.32 mL), 16 mmol of vinyl ester (vinyl hexanoate, vinyl octanoate, vinyl decanoate, or VL), 0.4 g of enzyme, and 0.4 g of molecular sieves at 70 °C with stirring at 125 rpm. The corresponding approximate initial liquid‐phase volumes were 3.88 mL for vinyl hexanoate, 4.42 mL for vinyl octanoate, 4.93 mL for vinyl decanoate, and 5.48 mL for VL.

After 6 h of reaction, the vial was removed from the 70 °C oil bath and allowed to cool to approximately 40 °C. A 10 mL portion of water was then added to the reaction mixture, which was homogenized by sonication for 30 min. The resulting turbid suspension was centrifuged at 8,000 rpm for 10 min, and the lower hydrophilic phase was collected. This phase was further centrifuged at 13,500 rpm for 3 min to remove residual hydrophobic impurities and solid particles. The resulting clear solution was serially diluted with water, with a total dilution factor of up to 3,000‐fold, to bring the glucose concentration within the calibration range. The concentration of unreacted glucose in the lower hydrophilic phase was quantified using a glucose oxidase/peroxidase enzymatic assay kit (GAGO20, Sigma–Aldrich, USA) according to the manufacturer's instructions [41]. An aliquot of the diluted sample (0.2 mL) was used for each assay, and the final assay volume was 1.0 mL. The glucose concentration in the original hydrophilic phase was calculated by applying the corresponding total dilution factor. Due to the lack of authentic standards for several glucose esters, the reaction rate was calculated using Equation (2), where the amount of formed product was replaced by the amount of consumed glucose.

### 2.8 Statistical Analysis

The results are expressed as mean ± standard deviation (*n* = 3). Statistical analysis was performed by one‐way analysis of variance followed by Tukey's test using IBM SPSS Statistics software (version 25). Differences were considered statistically significant at *p* < 0.05.

## 3 Results and Discussion

### 3.1 Reaction Network in RDES for Selective Glucose Ester Production

**SCHEME 1:**
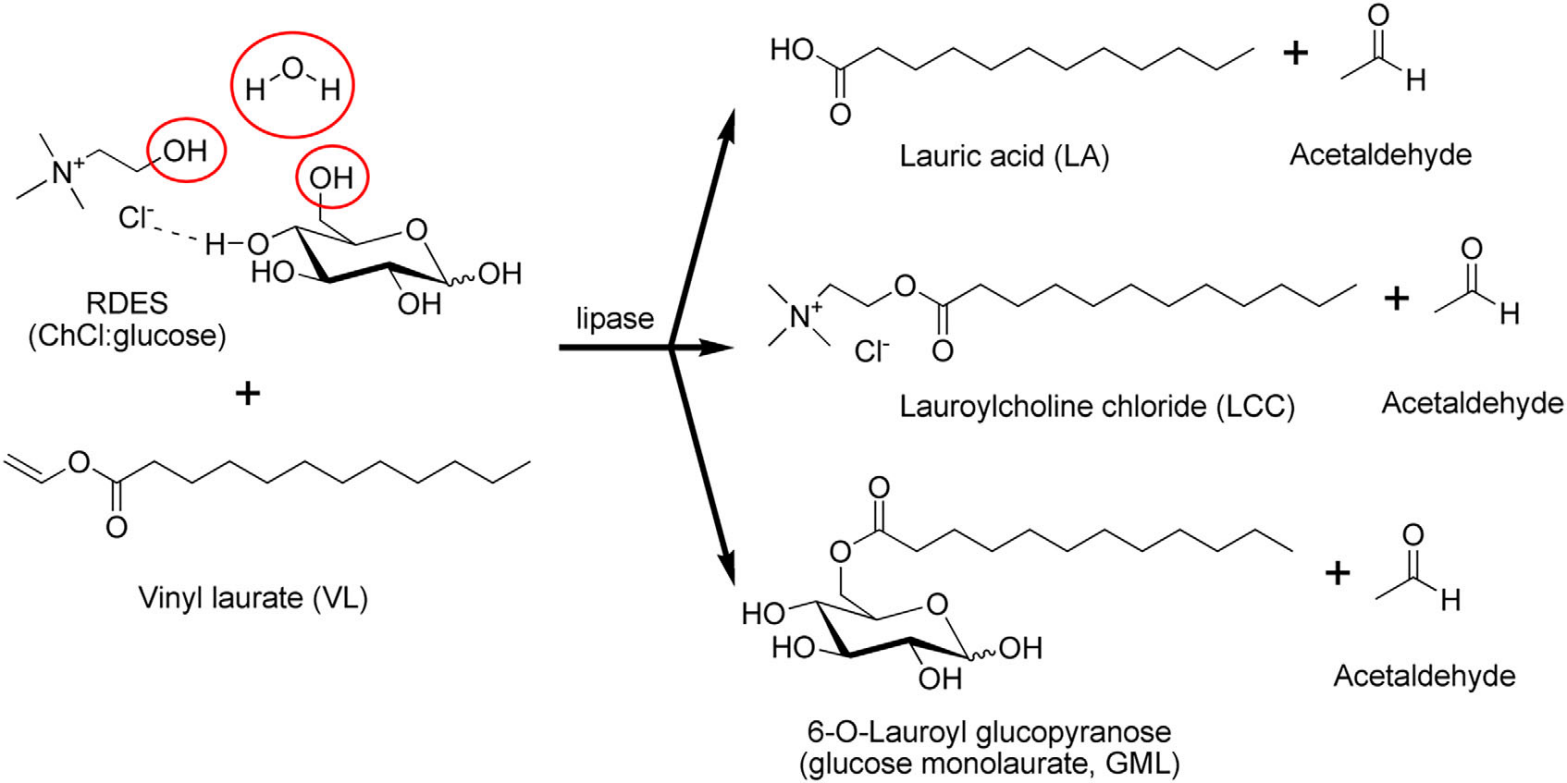
Lipase‐catalyzed glucose esterification with vinyl laurate and its competing hydrolytic and choline‐mediated transesterification pathways in a ChCl–glucose reactive deep eutectic solvent. The hydrogen bond depicted between ChCl and glucose represents a simplified example rather than a specific hydroxyl position.

In the ChCl–glucose RDES system used in this study, lipase‐catalyzed acyl transfer proceeds through a reaction network wherein glucose ester formation competes with multiple undesired pathways originating from a common acyl–enzyme intermediate (Scheme 1). The RDES comprises ChCl, glucose, and a small amount of water absorbed by both components, rendering choline, glucose, and water all capable of acting as nucleophiles during enzymatic catalysis.

Upon nucleophilic attack of the catalytic serine residue on VL, a lauroyl–enzyme intermediate is formed with concomitant release of vinyl alcohol, which rapidly and irreversibly tautomerizes to acetaldehyde, thereby driving the acylation step forward [42]. Subsequent deacylation of the lauroyl–enzyme intermediate can proceed via three competing pathways. When water acts as the nucleophile, hydrolysis occurs to yield LA, which shows substantially lower reactivity toward the subsequent direct esterification of glucose compared with that of vinyl ester‐mediated transesterification. It is therefore unfavorable for the target reaction [43]. Alternatively, transesterification with choline leads to the formation of LCC because choline contains a reactive hydroxyl group. Indeed, Buzatu et al. reported that in ChCl–glucose–water systems, lipase‐catalyzed reactions preferentially produce LCC rather than glucose ester, demonstrating the dominance of competing pathways under specific combinations of solvent composition and water content [38]. In contrast, the nucleophilic attack by glucose results in the formation of GML, the desired glucose ester product.

Because these pathways diverge from the same acyl–enzyme intermediate, product distribution is expected to depend on the relative rates of hydrolysis and competing transesterification reactions. Accordingly, controlling water content and the ChCl:glucose ratio provides practical means to minimize VL hydrolysis and choline‐derived ester formation while promoting glucose acylation, as examined in the following sections.

### 3.2 Effect of Water on Lipase‐Catalyzed Glucose Esterification in RDES

The effect of water content on the lipase‐catalyzed glucose esterification was investigated by introducing controlled amounts of water into the ChCl–glucose RDES (Figure 1). As the water content increased, the formation rate of GML decreased markedly, whereas the formation rate of LA increased and reached a plateau at water contents greater than 1.2 wt%. This opposite trend indicates that hydrolysis becomes increasingly favored relative to glucose esterification as water content increases. As water exhibits a higher nucleophilicity toward the acyl–enzyme intermediate [44], it preferentially attacks the lauroyl–enzyme intermediate, thereby accelerating hydrolytic deacylation relative to transesterification. Consistent with previous reports, increased water availability in low‐water systems enhances hydrolytic pathways relative to transesterification [45]. Consequently, enhanced conversion of VL to LA reduces the effective availability of the acyl donor for glucose‐mediated transesterification, leading to decreased glucose ester formation.

In contrast, the formation rate of LCC showed a different dependence on water content. At 0.2 wt% water, the LCC formation rate was among the lowest values observed and was not statistically different from those measured at higher water contents (4.2 and 5.2 wt%). As the water content increased to 1.2 wt%, the LCC formation rate increased markedly and remained relatively high between 1.2 and 2.2 wt%, followed by a gradual decrease at water contents above 3.2 wt%. Comparison of the formation rates of GML and LCC revealed that the difference between the two rates was greatest at 0.2 wt% water, where the formation of the desired GML proceeded substantially faster than that of LCC. In contrast, at 2.2 wt% water the formation rates of GML and LCC became most similar, indicating a reduced selectivity toward GML formation. This observation is partially consistent with the results reported by Buzatu et al., who showed that in a ChCl:glucose:water (2:1:1) RDES containing a relatively high water content, the LCC was obtained as the sole detectable product during lipase‐catalyzed reactions, although their system employed LA rather than VL as the acyl donor.

These results suggest that minimizing the initial water content is advantageous for achieving higher selectivity toward GML formation relative to the competing hydrolysis of VL and choline‐mediated transesterification pathways. Therefore, no water was intentionally added in the subsequent experiments, and molecular sieves were included to minimize residual and adventitious moisture. Nevertheless, LA formation could not be completely suppressed, indicating that a limited amount of catalytically available water remained in the reaction system despite the moisture‐control measures. In ChCl‐based DESs, water can participate in composition‐dependent interactions with chloride ions and the hydrogen‐bond network, and its molecular environment cannot be inferred from total water content alone [46]. Therefore, the molecular state and distribution of this residual water within the RDES could not be determined and require further investigation.

### 3.3 Effect of DES Composition on Lipase‐Catalyzed Glucose Esterification in RDES

To evaluate the effect of DES composition on lipase‐catalyzed glucose esterification, a series of ChCl–glucose RDESs with varying molar ratios, namely C2G1, C1G1, C1G2, C1G3, and C1G4, were prepared and characterized (Table 1 and Figure S5). Increasing the mole fraction of glucose aimed to enhance its availability as a competing nucleophile in the reaction network, but this compositional change simultaneously altered the key physicochemical properties of RDESs.

**TABLE 1:** Characteristics of RDESs comprising ChCl and glucose analyzed in this work.

Abbreviation	Molar ratio (ChCl:glucose)	Preparation time, min	Water content, %	Density, g/mL	Color	
C2G1	2:1	<20	<0.01	1.18	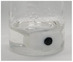	Colorless
C1G1	1:1	20	0.19	1.24	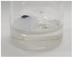	Colorless
C1G2	1:2	40	0.23	1.36	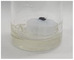	Slightly yellow
C1G3	1:3	90	1.99	1.37	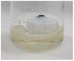	Slightly yellow
C1G4	1:4	120	ND^a^	1.37	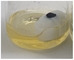	Yellow

a Not determined because the viscosity was too high.

As the mole fraction of glucose increased, the preparation time, water content, density, and degree of caramelization showed increasing trends, as indicated by color change. ChCl–glucose DESs readily absorb water owing to abundant hydrogen‐bonding functional groups, and the higher hydroxyl density of glucose further enhances water uptake at elevated glucose ratios [47]. Concomitantly, the density of RDESs increased from 1.18 to 1.36 g/mL as the glucose mole fraction increased from 0.33 to 0.66 and then approached a plateau of approximately 1.37 g/mL at higher glucose contents, reflecting increased molecular packing associated with higher glucose loading and saturation of the hydrogen‐bonding network at glucose‐rich compositions [29, 48]. From a practical and kinetic standpoint, excessive caramelization is undesirable because it reduces the fraction of chemically reactive glucose [49]. Accordingly, although higher glucose mole fractions increase substrate availability, the accompanying penalties for water uptake and caramelization outweigh these benefits at extreme compositions.

The thermal behavior of the ChCl–glucose mixtures was further investigated by DSC (Figure S5). During the second heating cycle, C2G1, C1G1, C1G2, C1G3, and C1G4 exhibited glass‐transition temperatures (*T*_g_) of −8.5, −34.0, −19.9, −4.7, and −6.5 °C, respectively. These glass transitions confirm the presence of an amorphous phase in all formulations, while no distinct crystallization or melting transitions attributable to crystalline glucose or ChCl were observed within the investigated temperature range. However, the absence of such transitions does not establish whether all glucose participates in the RDES hydrogen‐bond network, because DSC alone cannot distinguish network‐associated glucose from molecularly dissolved or weakly associated glucose within an amorphous phase [50]; therefore, the possibility that a fraction of the excess glucose was molecularly dissolved or weakly associated cannot be excluded. The variation in *T*_g_ among C2G1–C1G3 indicates that the ChCl‐to‐glucose ratio affected the bulk molecular mobility and intermolecular environment of the RDES phase. Notably, C1G3 and C1G4 exhibited similar *T*_g_ values, indicating that increasing the glucose content from C1G3 to C1G4 caused little further change in the bulk thermal behavior, although this similarity does not establish the molecular state of the excess glucose. Nevertheless, the high viscosity, longer preparation time, and more pronounced yellow coloration of C1G4 made it operationally unsuitable under the conditions used in this study. Accordingly, C1G4 was not included in the enzymatic reaction experiments, and C2G1, C1G1, C1G2, and C1G3 were selected for further evaluation.

**FIGURE 2:**
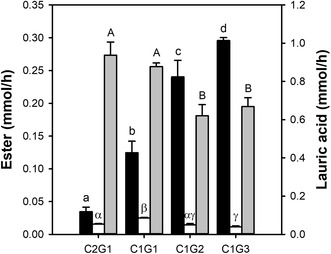
Reaction rates for glucose monolaurate (black bars) and lauroylcholine chloride (white bars) formation during the lipase‐catalyzed reaction of vinyl laurate in various ChCl–glucose RDESs. Formation rates of lauric acid (gray bars) are shown on the right axis. Reaction conditions: 8 mmol glucose; 8 mmol vinyl laurate; 0.4 g (4.7 U) enzyme; 0.4 g molecular sieve; 125 rpm; 70 °C; 6 h. Different letters indicate statistically significant differences (*p* < 0.05).

The product distribution obtained from the lipase‐catalyzed reaction of VL in the four selected ChCl–glucose RDESs reflects the kinetic competition among the three nucleophiles (water, choline, and glucose) for the acyl–enzyme intermediate (Figure 2). This compositional dependence is consistent with the reaction network outlined in Scheme 1. As the glucose mole fraction in the RDES increased, the formation of the target product (GML) increased markedly, whereas LA formation decreased, indicating suppression of hydrolysis. Specifically, the formation rate of GML increased from 0.035 mmol/h in C2G1 to 0.296 mmol/h in C1G3, whereas the formation rate of LA decreased from 0.938 to 0.668 mmol/h over the investigated compositions. The substantial increase in GML formation suggests that increased glucose availability contributed to enhanced glucose‐mediated acyl transfer at higher glucose mole fractions. However, no statistically significant difference in the formation rate of LA was observed between C1G2 and C1G3, which can be attributed to the higher water content of C1G3 counteracting the benefits of increased glucose availability. Similarly, LCC formation was lowest in C1G3, and the reduction in LCC formation rate relative to C2G1 was statistically significant. The LCC formation rate increased from C2G1 to C1G1 and subsequently decreased in C1G2 and C1G3, reaching its lowest value in C1G3. This result suggests that the highest glucose mole fraction tested reduced the relative contribution of choline‐mediated transesterification, likely because of the lower relative availability of choline as a competing nucleophile.

Collectively, these results indicate that pathway selectivity in ChCl–glucose RDESs reflects competition among water‐, glucose‐, and choline‐mediated reactions. The observed dependence on solvent composition is consistent with a kinetically influenced regime rather than simple thermodynamic equilibration [38, 51]. In addition to compositional effects, strong hydrogen‐bonding interactions within the DES matrix can influence substrate mobility and enzyme–substrate interactions within the reaction medium [37, 52]. Intentional distortion of the ChCl:glucose molar ratio thus serves a dual function: increasing the relative availability of glucose as a nucleophile while simultaneously modifying the hydrogen‐bonding network that may affect mass transfer characteristics [29]. Similar effects have been reported by Abbasi et al., who showed that deviations from stoichiometric HBD/HBA ratios reduce hydrogen‐bond acidity and reorganize the DES microstructure [53]. Based on this balance between enhanced glucose availability, manageable water content, and suppression of choline‐mediated ester formation, C1G3 was identified as the most suitable RDES composition for selective glucose esterification. Because glucose esterification, hydrolysis, and choline esterification proceed concurrently in this system, product distribution is governed primarily by relative reaction rates rather than thermodynamic equilibrium [54, 55]. Therefore, subsequent optimization focused on enhancing the reaction rate.

### 3.4 Effect of Glucose to VL Ratio on Lipase‐Catalyzed Glucose Esterification in RDES

In the optimization of the DES composition described above, the GML yield reached 22.3% in the C1G3 RDES, corresponding to approximately 1.77 mmol (0.64 g) of product formed within 6 h in a relatively small reaction volume (ca. 3.4 mL). Despite this favorable product formation, a substantial fraction of the glucose remained unreacted. Moreover, the recovery or recycling of substrates in the RDES system was hindered by the formation of a complex emulsion phase during the reaction. These limitations suggest the need for further optimization to improve substrate utilization and overall process efficiency.

**FIGURE 3:**
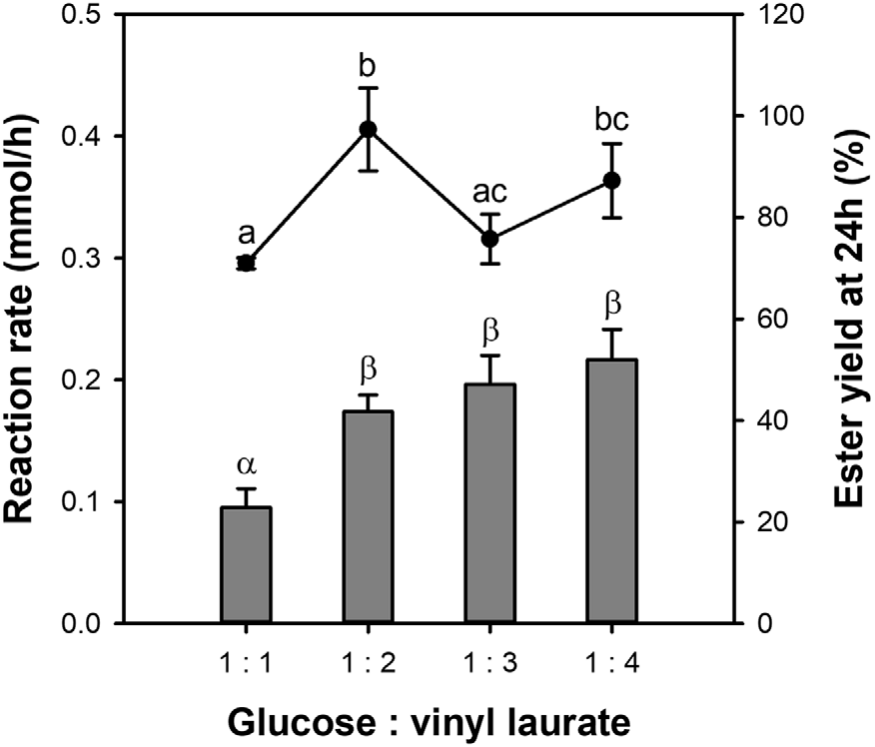
Reaction rate determined over the first 6 h (filled circles) and glucose monolaurate yield at 24 h (bars) during lipase‐catalyzed transesterification of glucose at different glucose‐to‐vinyl laurate ratios in C1G3. Reaction conditions: C1G3 RDES (2.67 mmol ChCl and 8 mmol glucose), 0.4 g (4.7 U) enzyme, 0.4 g molecular sieve, 125 rpm, 70 °C, 24 h. Different letters indicate statistically significant differences (*p* < 0.05).

As this reaction system is governed primarily by kinetic rather than thermodynamic factors, the effect of acyl donor loading was evaluated using the reaction rate (Figure 3). At a glucose:VL molar ratio of 1:1, the reaction rate was 0.30 mmol/h, which was insufficient to achieve a high reaction rate and product yield. Increasing the VL loading to a glucose:VL molar ratio of 1:2 gave the highest reaction rate (0.41 mmol/h), whereas further increases resulted in a decline, likely owing to unfavorable conditions caused by excessive acyl donor loading. Increasing the VL amount from 8 to 32 mmol expanded the hydrophobic VL phase from approximately 2.1 to over 8.3 mL, whereas the C1G3 RDES phase remained nearly constant at approximately 1.3 mL. In biphasic RDES–acyl donor systems, immobilized enzymes localize near the phase interface; therefore, excessive VL loading may increase the hydrophobic phase volume without proportionally enhancing enzyme–substrate contact.

The effect of acyl donor loading on GML yield after 24 h is also shown in Figure 3. The GML yield increased with an increasing VL molar ratio, reaching 22.8, 41.7, 47.2, and 52.0% at glucose:VL molar ratios of 1:1, 1:2, 1:3, and 1:4, respectively. However, the incremental increase in yield diminished at higher VL loadings. Moreover, when the yield was normalized to the amount of VL added, a continuous decrease in yield was observed with increasing VL content, indicating inefficient utilization of the acyl donor. Accordingly, interpretation based solely on the product yield can be misleading under these reaction conditions.

Based on the combined evaluation of the reaction rate and product yield, a glucose:VL molar ratio of 1:2 was identified as optimal. This ratio provides the most favorable balance between reaction rate and GML productivity while avoiding the economic and mechanical disadvantages associated with excessive acyl donor loading.

### 3.5 Effect of Enzyme Loading on Lipase‐Catalyzed Glucose Esterification in RDES

**FIGURE 4:**
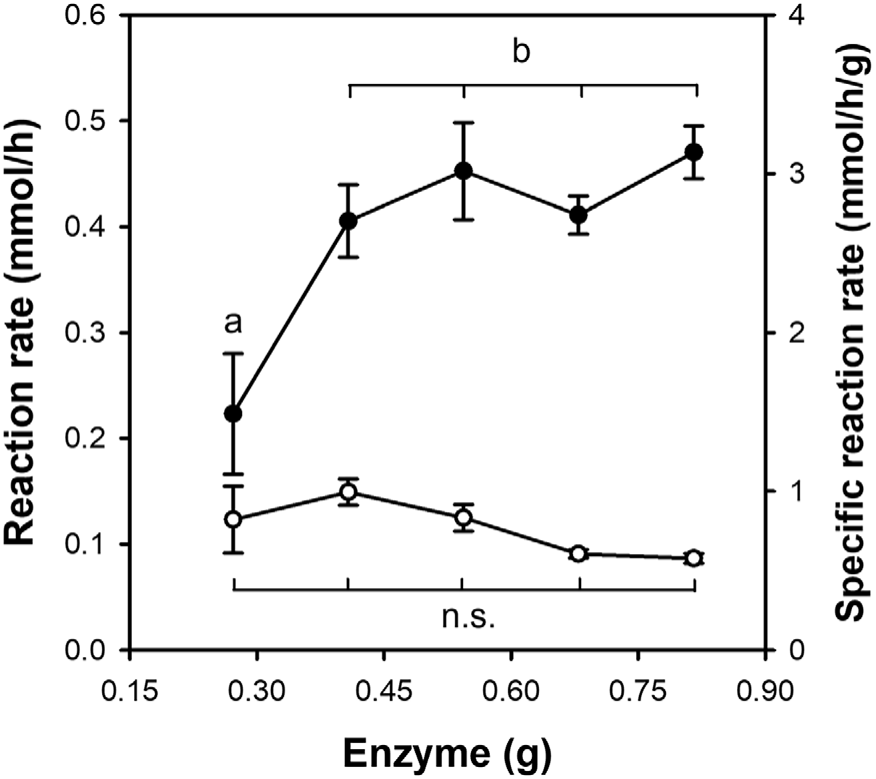
Reaction rate (filled circles) and specific reaction rate (unfilled circles) during the lipase‐catalyzed transesterification of glucose in C1G3 at different enzyme loadings. Novozym 435 loadings of 0.27, 0.40, 0.54, 0.68, and 0.82 g corresponded to approximately 3.2, 4.7, 6.4, 8.0, and 9.7 U, respectively. Reaction conditions: C1G3 RDES (2.67 mmol ChCl and 8 mmol glucose), 16 mmol vinyl laurate, 0.4 g molecular sieve, 125 rpm, 70 °C, 6 h. Different letters indicate statistically significant differences (*p* < 0.05). “n.s.” indicates no statistically significant difference (*p* > 0.05).

The effect of enzyme loading on glucose esterification was further investigated (Figure 4). As the enzyme content increased, the reaction rate increased from 0.22 mmol/h at 0.27 g to 0.47 mmol/h at 0.82 g. However, beyond an enzyme loading of 0.4 g, further increases resulted in only marginal improvements, and the reaction rate approached a plateau, with no statistically significant differences observed among higher enzyme loadings.

To identify the most productive and cost‐effective operating conditions, the reaction rate was normalized to the mass of enzyme used. The specific reaction rate (mmol/h/g) increased to an enzyme loading of 0.4 g but decreased at higher loadings, indicating diminishing returns under conditions of excessive enzyme addition. However, these differences were not statistically significant (*p* > 0.05). This behavior suggests that increasing the enzyme content beyond a certain level does not proportionally enhance catalytic efficiency when substrate availability is fixed [56]. In biphasic RDES–acyl donor systems, the effective reaction zone is expected to be limited, and excessive enzyme loading may thus fail to proportionally increase the effective enzyme–substrate interactions. Further, higher enzyme loading inevitably introduces a larger amount of enzyme‐associated water into the system. Even trace amounts of such localized water in the vicinity of the enzyme‐active sites may promote competing hydrolytic reactions of VL, thereby diminishing the apparent benefit of excessive enzyme addition. Accordingly, an enzyme loading of 0.4 g was selected as the optimal condition, as it represented the lowest enzyme amount among the statistically indistinguishable conditions, while maintaining both a high reaction rate and a favorable reaction rate per unit enzyme mass.

### 3.6 Structural Confirmation of the Purified Glucose Ester

To confirm the formation of GML, the purified product was analyzed by ESI‐MS using a linear ion trap mass spectrometer (Figure S3 and Table S1). GML has a calculated molecular mass of 362.5 Da, and the signal at *m*/*z* 385.4 was assigned to the sodium‐adducted molecular ion [M + Na]^+^. An additional ion at *m*/*z* 747.1 was assigned to the noncovalent sodium‐adducted dimer [2M + Na]^+^, a common feature in electrospray ionization arising from intermolecular association in solution [57, 58]. Sodium adduct formation is frequently observed in ESI‐MS [59]. A peak at *m*/*z* 286.4 was assigned to an LCC‐derived ion, consistent with chloride loss under ionization conditions. Similar sodium adducts of sugar esters and choline‐derived side products have been reported for esterification reactions in ChCl‐based DESs, as analyzed by ESI‐QTOF MS [35]. Although the LCC‐derived signal was relatively intense in the MS spectrum, LCC was detected only at trace levels by HPLC and NMR, likely reflecting differences in ionization response and solubility in the MS solvent.

The ^1^H and ^13^C NMR spectra of the purified product (Figure S4) were consistent with previously reported data for 6‐O‐lauroyl glucose [60, 61]. Signals corresponding to the lauryl chain, glucose moiety, and ester carbonyl carbon at 173.0 ppm confirmed ester formation [61, 62]. The downfield shifts of the C‐6 methylene protons and the corresponding C‐6 carbon signal supported predominant esterification at the primary hydroxyl group of glucose [63, 64]. Together, the MS and NMR results confirmed the product as 6‐O‐lauroyl glucose.

### 3.7 Performance Comparison Between RDES and a Conventional Organic Solvent System for Lipase‐Catalyzed Glucose Esterification

The optimized RDES‐based reaction system was compared with a conventional organic solvent‐based system. Among organic solvents, 2M2B is one of the most widely reported media for the lipase‐catalyzed esterification of sugars, as it provides adequate enzyme stability while maintaining moderate sugar solubility. In many studies, 2M2B was supplemented with 20% (v/v) DMSO as a cosolvent to enhance sugar dissolution [65]. To enable a fair comparison of solvent efficiency, all reaction parameters—including initial substrate loading, enzyme and molecular sieve contents, reaction temperature, and total reaction volume—were maintained identical in both systems, while stirring speed was adjusted to ensure stable phase mixing in each medium. In particular, a higher stirring speed (150 rpm) was applied in the 2M2B/DMSO system to facilitate dispersion of undissolved glucose, whereas 125 rpm was sufficient for the homogeneous RDES system. It should be noted that, despite identical initial glucose loading, the effective dissolved glucose concentration differed substantially between the two systems (Table 2), owing to solubility limitations in the organic solvent system.

**TABLE 2:** Volumetric productivity of lipase‐catalyzed glucose esterification in various DES and organic solvent systems.

Reaction medium	Temp., °C	Enzyme	Acyl source	Glucose content, g/L	Productivity, g L^−1^ h^−1^	Ref.
ChCl:glycerol (1:2 molar ratio)	37	Porcine pancreatic lipase	VL	5.0 (5.0)^a^	0.41	[66]
ChCl:glycerol(1:2)/DMSO/2M2B (1/1/3 vol. ratio)	50	Lipomode 768	VL	32.5 (32.5)	0.34	[67]
Menthol:LA (2:1 molar ratio)	70	Novozym 435	VL, LA	22.7 (<0.3)	0.88	[68]
2M2B/DMSO (4/1 vol. ratio)	70	Novozym 435	VL	263.9 (20.2)	1.32	This study
C1G3 RDES	70	Novozym 435	VL	263.9 (263.9)	12.04^b^	This study

a Values in parentheses indicate the dissolved glucose content under the reported conditions.

b Productivity was calculated based on the maximum yield obtained after 21 h of reaction.

**FIGURE 5:**
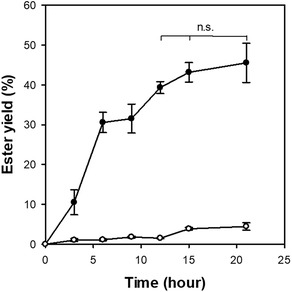
Time course for lipase‐catalyzed transesterification of glucose with vinyl laurate in the RDES system (filled circles) and the organic solvent system (unfilled circles). Reaction conditions for the RDES system: C1G3 RDES (2.67 mmol ChCl and 8 mmol glucose); 16 mmol vinyl laurate; 0.4 g (4.7 U) enzyme; 0.4 g molecular sieve; 125 rpm; 70 °C. Reaction conditions for the organic solvent system: 2‐methyl‐2‐butanol 1.3 mL (with 20 vol% DMSO); 8 mmol glucose; 16 mmol vinyl laurate; 0.4 g (4.7 U) enzyme; 0.4 g molecular sieve; 150 rpm; 70 °C. “n.s.” indicates no statistically significant difference (*p* > 0.05).

The time‐course profiles of GML formation in the two solvent systems over 21 h were compared (Figure 5). During the early stage of the reaction, transesterification in the RDES system proceeded rapidly, reaching a yield of 31% at 6 h, as reflected by the steep initial increase in GML concentration. This rapid progression was accompanied by the formation of an emulsion phase, which was observed only under high‐yield conditions. Although the interfacial characteristics were not quantified, the emergence of the emulsion was likely associated with the accumulation of the produced ester and may have contributed to enhanced interfacial interactions within the biphasic system [69]. The GML yield reached approximately 40% within the first 12 h. With prolonged reaction time, the yield increased slightly to about 46% at 21 h; however, this increase was not statistically significant.

In contrast, the GML yield obtained in the 2M2B/DMSO system after 21 h was only 4%, which is approximately one‐tenth of that achieved in the RDES system. This lower yield likely resulted from the combined effects of limited glucose solubility and time‐dependent enzyme deactivation in the 2M2B/DMSO system. To evaluate the operational stability of Novozym 435 under the organic solvent conditions, the enzyme was preincubated for 0, 3, or 6 h under the conditions described for the organic solvent system in Figure 5, except that glucose and VL were omitted during preincubation. After preincubation, glucose and VL were added, and GML synthesis was conducted for 3 h under the conditions described in Figure 5. The GML concentration was then quantified, and the formation rate was calculated from the amount of GML produced during this 3‐h reaction period. When the GML formation rate without preincubation was normalized to 100%, the relative rates after 3 and 6 h of preincubation decreased to 82% and 51%, respectively. This time‐dependent decrease indicates a substantial loss of catalytic performance in the 2M2B/DMSO system. Although 20% (v/v) DMSO improved glucose dissolution, the effective dissolved glucose concentration remained substantially lower than that in the RDES system, while further increasing the DMSO content may impair enzyme activity [70]. Furthermore, although DMSO can improve substrate solubility and reactivity, its use complicates downstream product purification. Consequently, the limited performance of the 2M2B/DMSO system should be attributed to both low effective glucose availability and insufficient enzyme stability under the tested conditions. In contrast, GML continued to accumulate in the RDES system during prolonged reaction, suggesting more favorable operational compatibility with Novozym 435. Overall, these results demonstrate that the RDES system—which accommodates a high concentration of the target substrate—offers substantially superior performance for lipase‐catalyzed glucose esterification compared to conventional organic solvent systems.

### 3.8 Comparison of Volumetric Productivity With Previously Reported DES and Organic Solvent Systems for Glucose Esterification

To further evaluate the relative performance of the newly developed RDES‐based system, its volumetric productivity was compared with that of previously published DES‐based lipase‐catalyzed glucose esterification systems (Table 2). This comparison includes studies employing both hydrophilic and hydrophobic DESs, which have been explored as reaction media and, in some cases, as reactive components. Despite differences in DES composition, many previously reported systems operate with relatively low effective glucose concentrations due to limited solubility, which may constrain volumetric productivity under their respective experimental conditions.

For example, Noro et al. reported near‐complete conversion of the supplied glucose in a hydrophilic DES (ChCl:glycerol of 1:2 molar ratio) system [66]. However, because the system relied on externally added glucose at a relatively low concentration, the absolute product amount and volumetric productivity were limited under the reported experimental conditions, likely reflecting substrate solubility constraints. A similar limitation was observed in the study by Hoyos et al., where a DES (ChCl:glycerol of 1:2 molar ratio)‐based cosolvent system combined with organic solvents formed a low‐viscosity emulsion but still exhibited modest productivity due to restricted substrate loading [67]. In contrast, Qi et al. proposed a hydrophobic RDES composed of LA, achieving a higher volumetric productivity of 0.88 g L^−1^ h^−1^ compared with that of hydrophilic DES systems [68]. Nevertheless, the overall product formation in that system was again constrained by the inherently very low solubility of glucose in the hydrophobic reaction medium.

In contrast, the RDES system developed in this study achieved a volumetric productivity of 17.3 g L^−1^ h^−1^, which is higher than those reported for several DES‐ and organic solvent‐based systems under comparable temperature and substrate loading conditions. This productivity was calculated based on the yield (40%) obtained after 12 h of reaction. Even when the productivity is calculated using the final yield (46%) obtained after 21 h, a value of 12.0 g L^−1^ h^−1^ is obtained, which still represents a markedly higher productivity than previously reported systems. This enhancement may be attributed, at least in part, to the ability of the ChCl–glucose RDES to maintain glucose in a highly concentrated and homogeneous state, thereby increasing the effective substrate availability for lipase‐catalyzed transesterification. In contrast to systems where glucose solubility limits the dissolved substrate concentration, the homogeneous nature of the ChCl–glucose RDES allows higher effective glucose availability. In addition, the use of molecular sieves to minimize water content and adjustment of the RDES composition reduced competing hydrolysis and choline‐mediated ester formation, thereby contributing to higher reaction rates and volumetric productivity.

From a sustainability perspective, the principal advantage of the present RDES system lies in process intensification rather than in the use of RDES alone. Because glucose serves as both a substrate and a component of the reaction medium, high substrate loading can be achieved without an additional bulk organic reaction solvent. Combined with the high volumetric productivity, this feature may reduce the reactor volume and processing time required for a given amount of product. The EtAc extraction and n‐hexane washing used in this study were employed for laboratory‐scale purification of high‐purity GML; therefore, future process development may allow reduced solvent consumption when such a high degree of product purity is not required. Further development should focus on simplified product recovery, solvent recycling, and enzyme and RDES reuse.

### 3.9 Effect of Acyl Chain Length on Lipase‐Catalyzed Glucose Esterification in RDES

**FIGURE 6:**
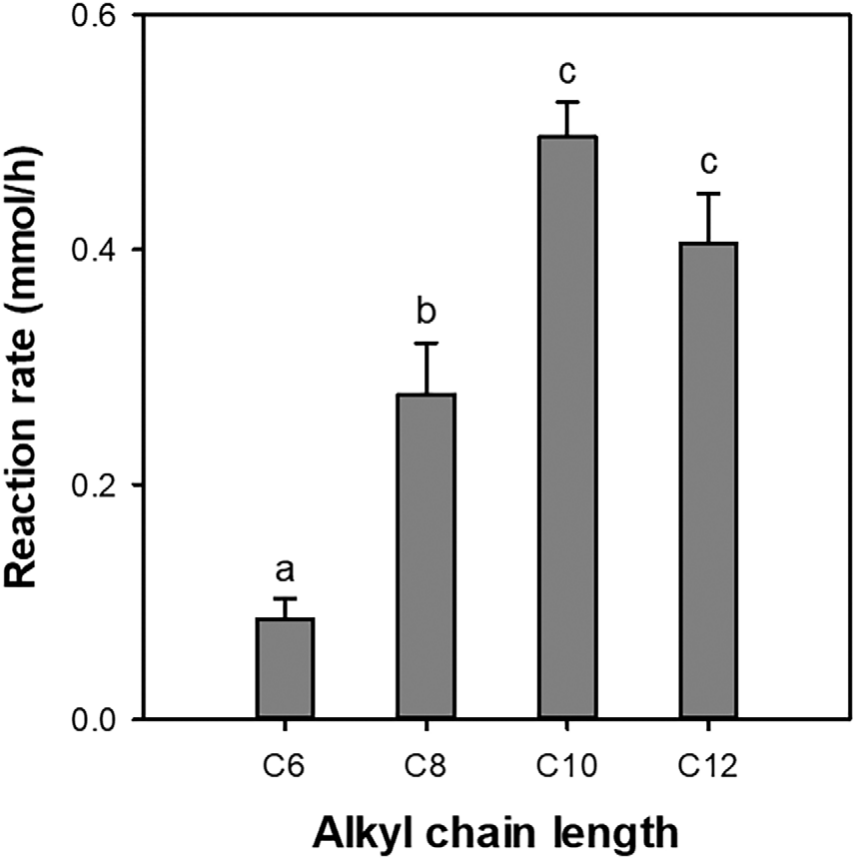
Reaction rates of lipase‐catalyzed transesterification of glucose in C1G3 using vinyl esters with different alkyl chain lengths. Reaction conditions: C1G3 RDES (2.67 mmol ChCl and 8 mmol glucose); 16 mmol vinyl ester (vinyl hexanoate (C6), vinyl octanoate (C8), vinyl decanoate (C10), or vinyl laurate (C12)), 0.4 g (4.7 U) enzyme, 0.4 g molecular sieve, 125 rpm, 70 °C, 6 h. Reaction rates were calculated based on glucose consumption due to the lack of authentic standards for the corresponding glucose esters. Different letters indicate statistically significant differences (*p* < 0.05).

To evaluate the versatility of the established RDES‐based glucose esterification system, vinyl esters with different alkyl chain lengths (vinyl hexanoate (C6), vinyl octanoate (C8), vinyl decanoate (C10), and VL (C12)) were examined as acyl donors under optimized reaction conditions. The reaction rates, calculated based on glucose conversion within the first 6 h, are summarized in Figure 6.

All tested vinyl esters successfully participated in the lipase‐catalyzed transesterification reaction, confirming that the present RDES system is not limited to a specific acyl donor. However, clear differences in reaction rates were observed depending on acyl chain length. The lowest reaction rate was obtained with vinyl hexanoate (0.085 mmol/h), whereas vinyl decanoate showed the highest rate (0.496 mmol/h), followed by VL and vinyl octanoate. Nevertheless, the difference between C10 and C12 was not statistically significant (*p* > 0.05). These results indicate that the reaction efficiency is influenced by the acyl donor structure, reflecting the intrinsic chain‐length selectivity of the lipases [71].

The observed reactivity trend differed from those reported for conventional organic solvent systems. For example, Arcens et al. reported a reactivity order of C6 > C10 > C12 > C8 in glucose esterification using acetonitrile as the reaction medium [43], whereas Soultani et al. observed reduced conversion with decreasing fatty acid chain length during fructose esterification in 2M2B [72]. Such discrepancies highlight that acyl donor reactivity is not solely determined by chain length but is also modulated by the reaction medium. In the present RDES system, the highly polar hydrogen‐bonded environment and biphasic nature of the reaction are expected to affect substrate partitioning, interfacial accessibility, and enzyme–substrate interactions, thereby reshaping the apparent chain‐length preference of the lipase. Indeed, enzyme reactivity and selectivity depend on the DES composition, which can modulate the local microenvironment surrounding the enzyme rather than acting as an inert solvent [26, 31].

## 4 Conclusion

The principal advance of this study is the demonstration that RDES formulation can be used as a kinetic control strategy for directing competing acyl‐transfer pathways in enzymatic glucose esterification. By adjusting the molar ratio of ChCl to glucose and controlling water content, the kinetic competition among water‐, choline‐, and glucose‐mediated pathways was shifted toward selective GML formation, while undesired hydrolysis and choline‐derived ester formation were suppressed.

The glucose‐rich ChCl–glucose RDES (C1G3) functioned as a reaction medium with high effective glucose availability, enabling efficient transesterification under nonaqueous conditions. Under optimized conditions, a GML yield of ~46% was achieved with a volumetric productivity of 12 g L^−1^ h^−1^, clearly exceeding that of a conventional 2M2B/DMSO system. The product was purified and confirmed as 6‐O‐lauroyl glucose by HPLC, MS, and ^1^H/^13^C NMR spectroscopy, and the reaction system was applicable to vinyl esters with different acyl chain lengths (C6–C12).

These findings establish engineered sugar‐based RDESs as kinetically active reaction platforms in which solvent composition can be used to tune pathway competition while maintaining high substrate availability. Compared with the conventional 2M2B/DMSO system, the C1G3 RDES avoided DMSO during catalysis, supported sustained product formation, and achieved substantially higher volumetric productivity, potentially reducing the downstream burden associated with removing a high‐boiling, broadly miscible cosolvent. Further studies are required to address process‐level sustainability and practical scale‐up, including the high viscosity of ChCl–glucose RDESs, energy requirements, solvent recovery or replacement, enzyme and RDES reusability, and downstream purification of glucose esters.

## Funding

This work was supported by Ministry of Oceans and Fisheries (RS‐2022‐KS221555); National Research Foundation of Korea (RS‐2026‐25488262); Ministry of Climate, Energy and Environment.

## Conflicts of Interest

The authors declare no conflicts of interest.

## Supporting information

Supplementary material

## Data Availability

The data that support the findings of this study are available from the corresponding author upon reasonable request.
