# Supplementary material for: Selective Enzymatic Synthesis of Glucose Fatty Acid Esters in a Choline Chloride–Glucose Reactive Deep Eutectic Solvent
Source: ChemSusChem. 2026 Aug 16;19(16):e70963. doi: 10.1002/cssc.70963 (PMC13478347; doi:10.1002/cssc.70963)
Supplement: Supplementary file 1 [file CSSC-19-e70963-s001.pdf]

Supporting Information (SI)

Selective Enzymatic Synthesis of Glucose Fatty Acid Esters in a Choline Chloride–Glucose Reactive Deep Eutectic Solvent

Jeong Eun Cha[1], Dojin Kim[1], Dong Han Kim[1], Keehoon Won[2,*], and Sang Hyun Lee[1,*]

[1] *Advanced Materials Program, Department of Biological Engineering, Konkuk University, Seoul 05029, Republic of Korea*

[2] *Department of Chemical and Biochemical Engineering, Dongguk University-Seoul, Seoul 04620, Republic of Korea*

*Corresponding authors:

Prof. Keehoon Won

Tel.: +82-2-2260-8922

E-mail: keehoon@dongguk.edu

Prof. Sang Hyun Lee

Tel.: +82-2-2049-6269

E-mail: sanghlee@konkuk.ac.kr

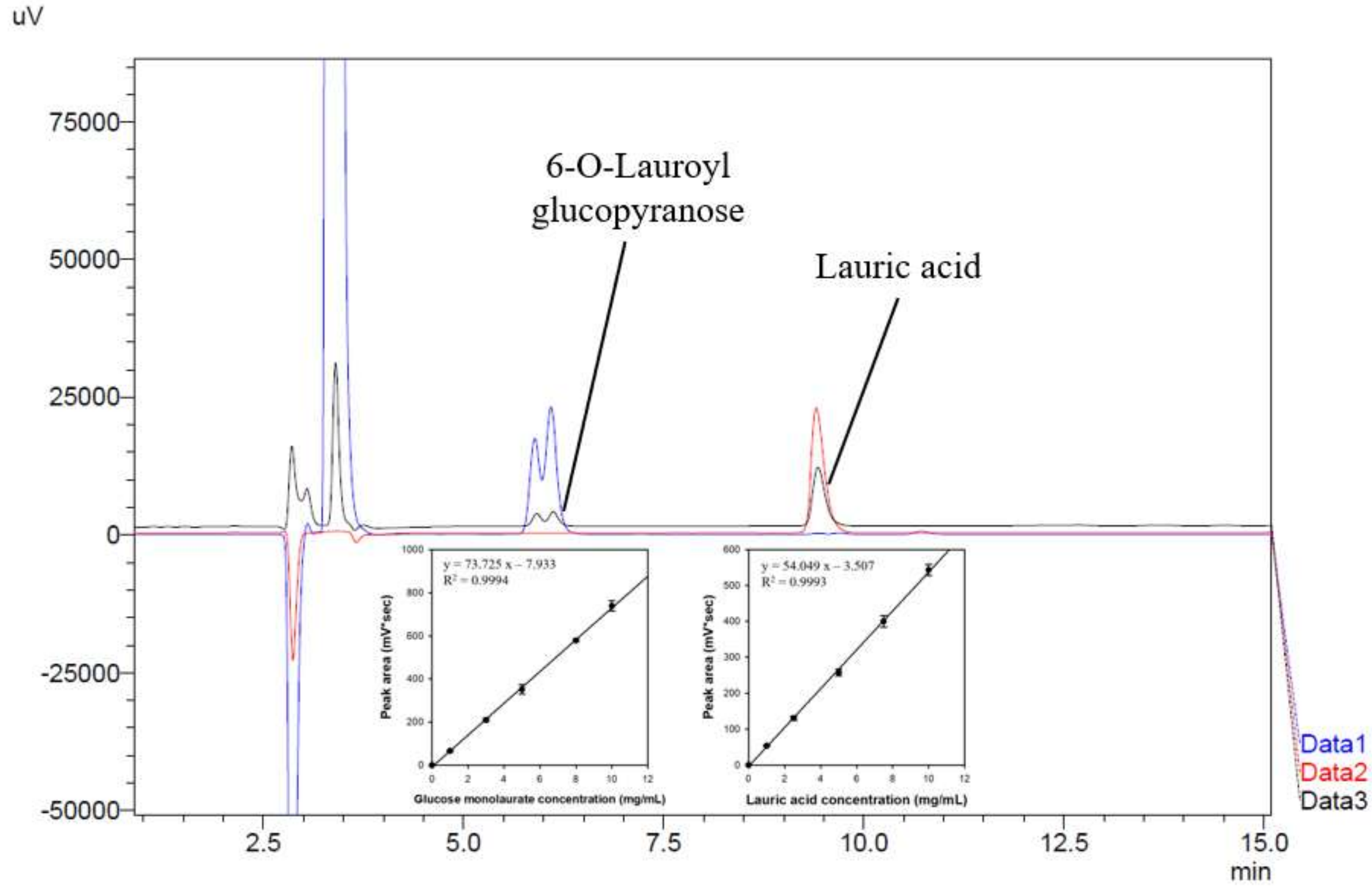

Figure S1. HPLC chromatograms of reacted mixture of the lipase-catalyzed transesterification in RDES (black trace), purified 6-O-lauroyl glucose (GML, blue trace) and an authentic lauric acid standard (LA, red trace). Reaction conditions: C1G3 RDES (2.67 mmol choline chloride and 8 mmol glucose); 16 mmol vinyl laurate; 0.4 g (4.7 U) enzyme; 0.4 g molecular sieve; 125 rpm; 70 °C; 6 h. The peak observed at approximately 3 min in the purified ester chromatogram corresponds to tetrahydrofuran, which was used to dissolve ester. The insets show the external calibration curves constructed using purified GML and authentic LA standards.

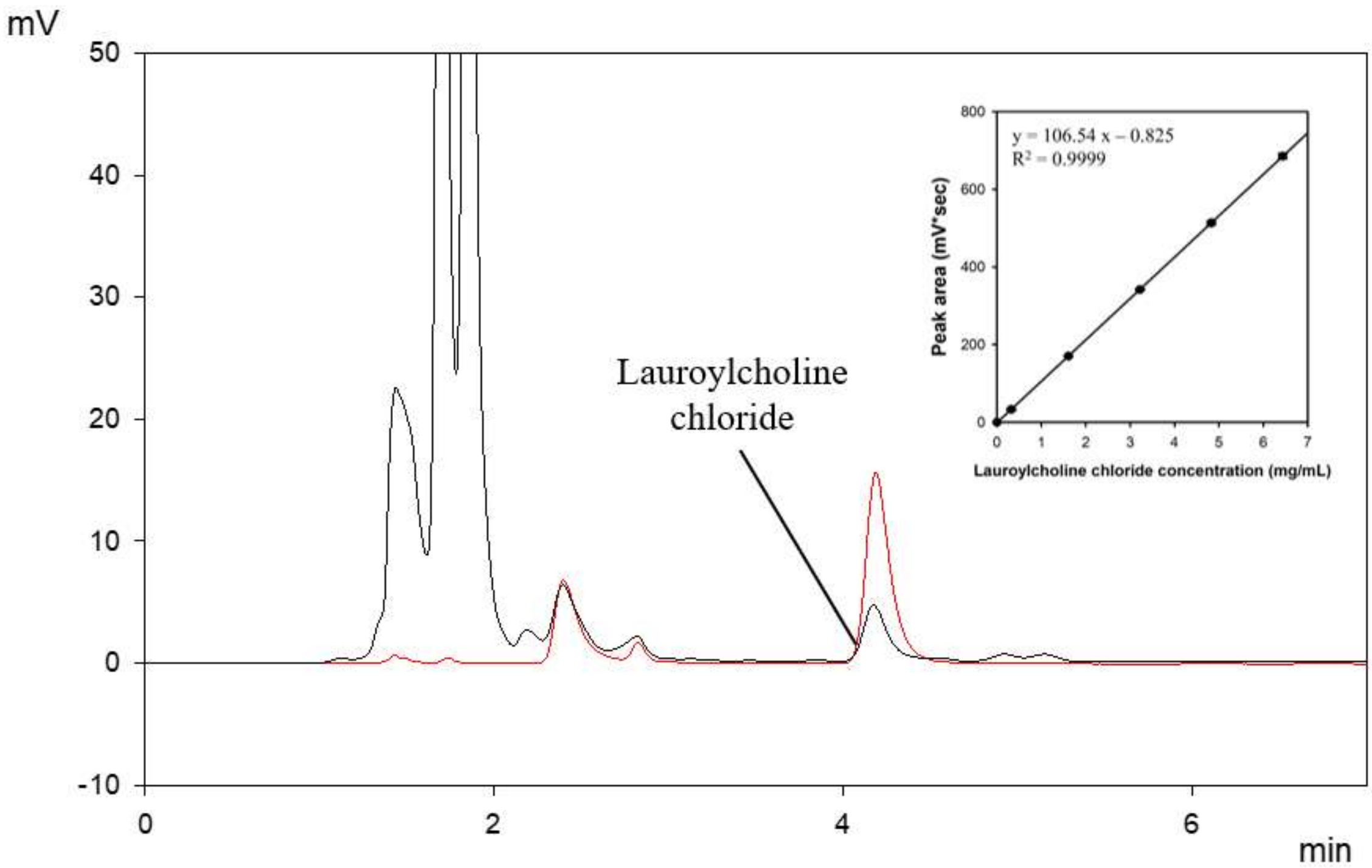

Figure S2. HPLC chromatograms of reacted mixture of the lipase-catalyzed transesterification in RDES (black trace) and an authentic lauroylcholine chloride standard (LCC, red trace). Reaction conditions: C2G1 RDES (16 mmol choline chloride and 8 mmol glucose); 8 mmol vinyl laurate; 0.4 g (4.7 U) enzyme; 0.4 g molecular sieve; 125 rpm; 70 °C; 6 h. The inset shows the external calibration curve constructed using authentic LCC standards.

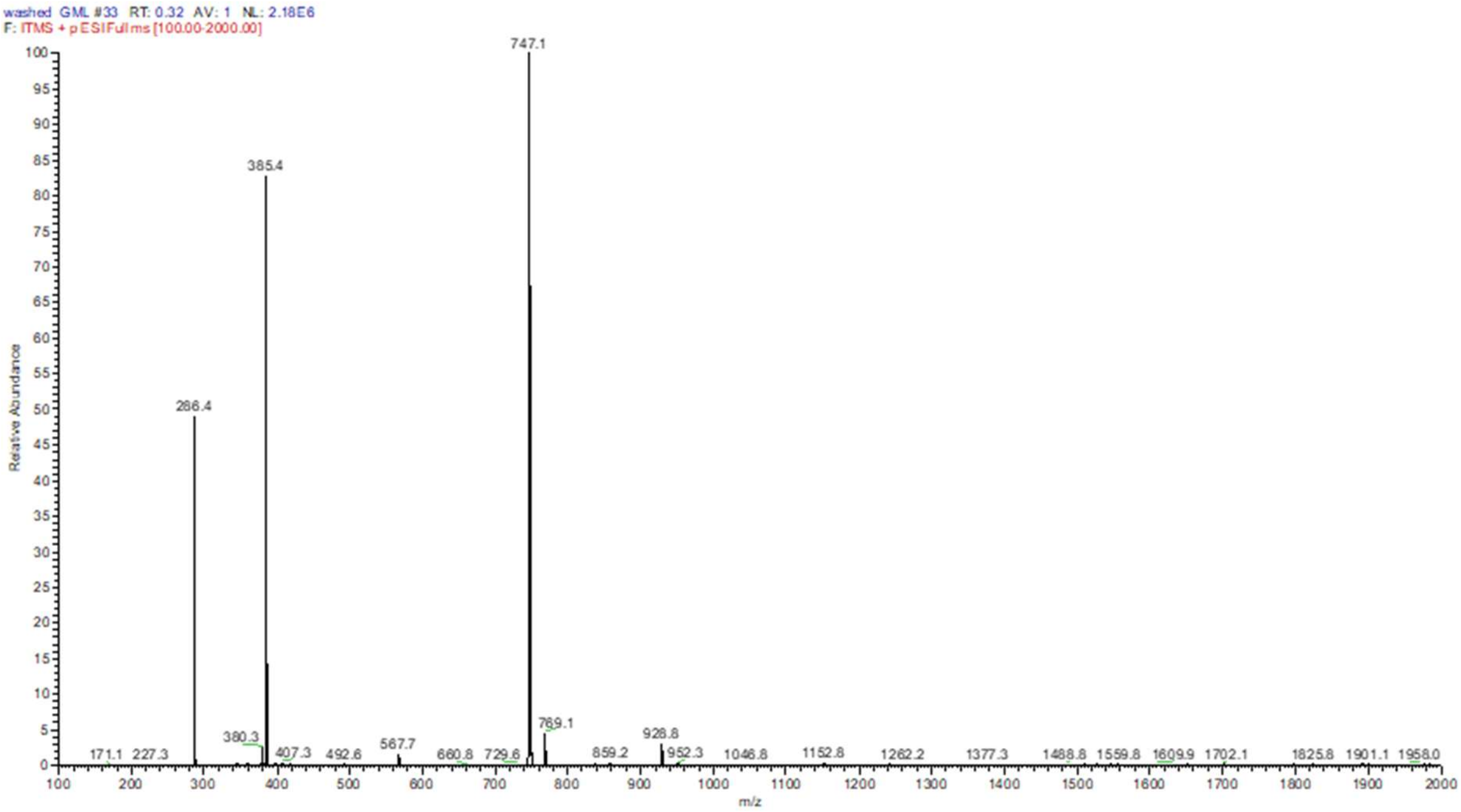

Figure S3. Mass spectrum of purified 6-O-lauroyl glucose (glucose monolaurate).

Table S1. Major ions observed in mass spectrum of purified 6-O-lauroyl glucose.

m/z value	Possible correspondent
286.4	$[\ LCC - Cl\ ]^{+}$
385.4	$[\ M + Na\ ]^{+}$
747.1	$[\ 2M + Na\ ]^{+}$

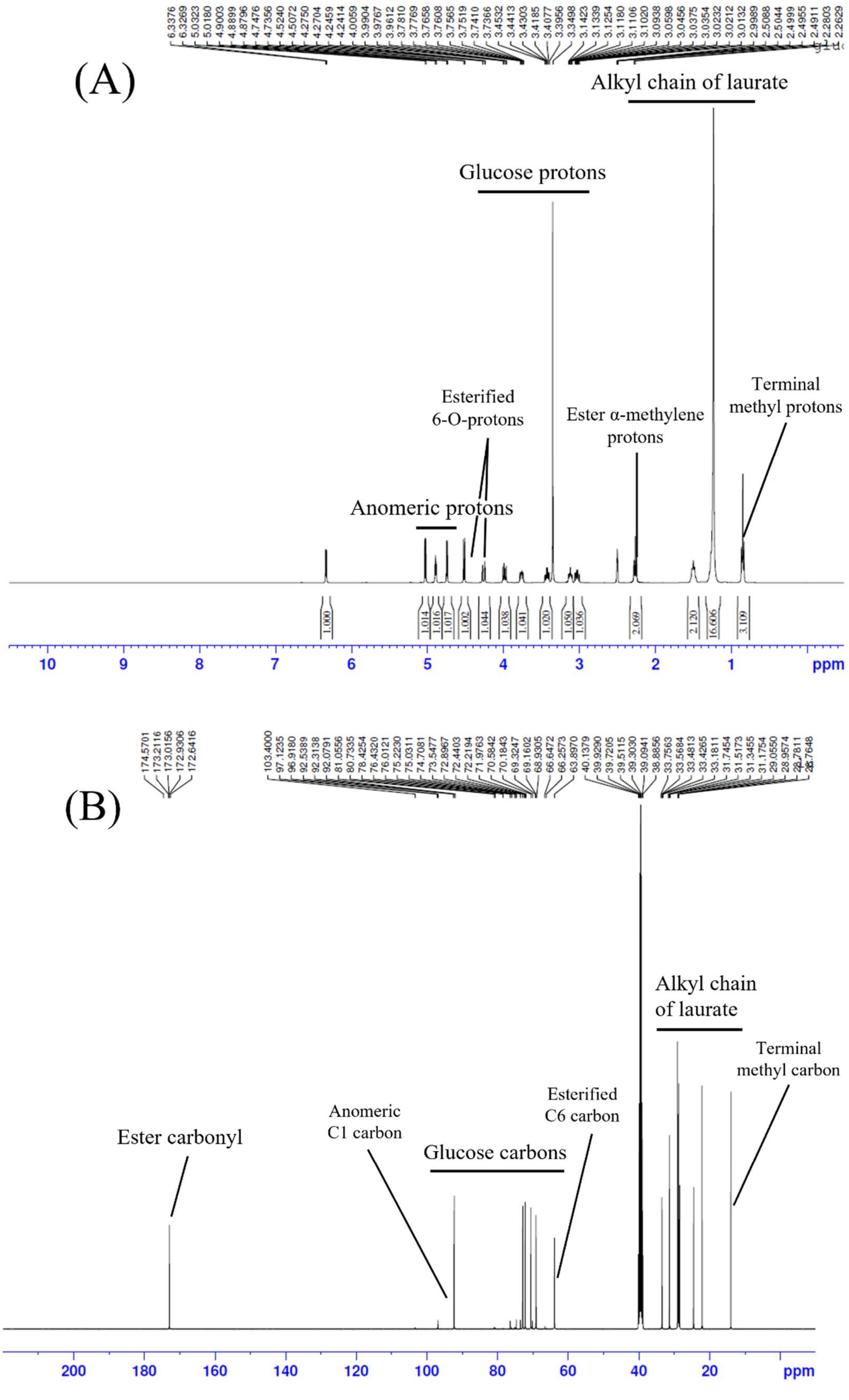

Figure S4. ^{1}H NMR spectrum (A) and ^{13}C NMR spectrum (B) of 6-O-lauroyl glucose.

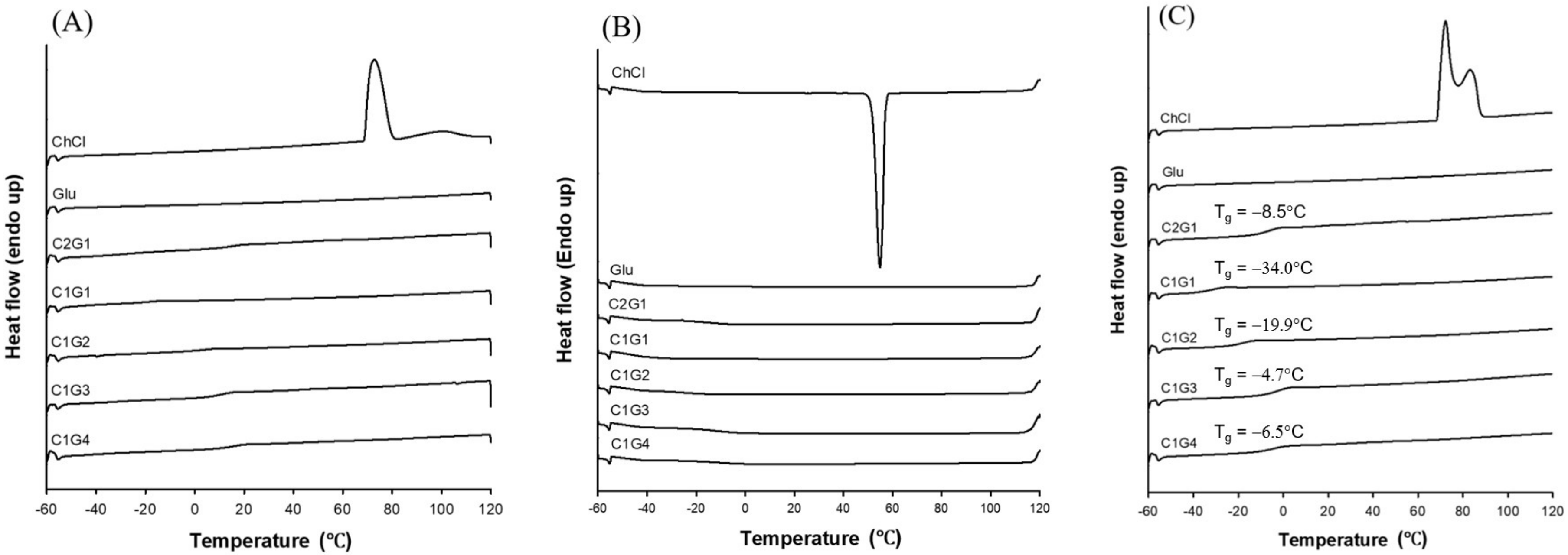

Figure S5. DSC thermograms of ChCl, glucose, and ChCl:glucose RDESs with different molar ratios obtained during (A) first heating, (B) cooling, and (C) second heating. The thermograms were vertically offset for clarity. Endothermic heat flow is upward (thermograms not to scale).
